# Understanding the Borderline Brain: A Review of Neurobiological Findings in Borderline Personality Disorder (BPD)

**DOI:** 10.3390/biomedicines13071783

**Published:** 2025-07-21

**Authors:** Eleni Giannoulis, Christos Nousis, Ioanna-Jonida Sula, Maria-Evangelia Georgitsi, Ioannis Malogiannis

**Affiliations:** 1 Laboratory of Psychometrics and Neuropsychology, Eginition Hospital, Medical School of Athens, 11528 Athens, Greece; 2 Specific Sector of Personality Disorders, Eginition Hospital, 11528 Athens, Greece; 3 The Model Community Center for Non-Suicidal Self-Injury in Young People, 11633 Athens, Greece

**Keywords:** borderline personality disorder, functional connectivity, default mode network, machine learning, fMRI, transdiagnostic, psychodynamic therapy, biomarkers

## Abstract

Borderline personality disorder (BPD) is a complex and heterogeneous condition characterized by emotional instability, impulsivity, and impaired regulation of interpersonal relationships. This narrative review integrates findings from recent neuroimaging, neurochemical, and treatment studies to identify core neurobiological mechanisms and highlight translational potential. Evidence from 112 studies published up to 2025 is synthesized, encompassing structural MRI, resting-state and task-based functional MRI, EEG, PET, and emerging machine learning applications. Consistent disruptions are observed across the prefrontal–amygdala circuitry, the default mode network (DMN), and mentalization-related regions. BPD shows a dominant and stable pattern of hyperconnectivity in the precuneus. Transdiagnostic comparisons with PTSD and cocaine use disorder (CUD) suggest partial overlap in DMN dysregulation, though BPD-specific traits emerge in network topology. Machine learning models achieve a classification accuracy of 70–88% and may support the tracking of early treatment responses. Longitudinal fMRI studies indicate that psychodynamic therapy facilitates the progressive normalization of dorsal anterior cingulate cortex (dACC) activity and reductions in alexithymia. We discuss the role of phenotypic heterogeneity (internalizing versus externalizing profiles), the potential of neuromodulation guided by biomarkers, and the need for standardized imaging protocols. Limitations include small sample sizes, a lack of effective connectivity analyses, and minimal multicenter cohort representation. Future research should focus on constructing multimodal biomarker panels that integrate functional connectivity, epigenetics, and computational phenotyping. This review supports the use of a precision psychiatry approach for BPD by aligning neuroscience with scalable clinical tools.

## 1. Introduction

### 1.1. Background

Borderline personality disorder (BPD) is a chronic and debilitating psychiatric condition characterized by pervasive patterns of emotional instability, disturbed identity, impulsive behavior, and dysfunctional interpersonal relationships [1]. It is associated with high rates of comorbidity, functional impairment, and suicidal behavior, with up to 10% of individuals ultimately dying by suicide [2]. BPD affects approximately 1.6% of the general population and up to 20% of psychiatric inpatients, making it one of the most commonly diagnosed personality disorders. Table 1 provides a summary of prevalence estimates across populations.

Traditionally, BPD has been conceptualized within psychodynamic and behavioral frameworks, with a particular focus on early attachment disruption, trauma, and maladaptive interpersonal schemas [8,9]. However, over the past two decades, neurobiological research has revealed various structural, functional, and neurochemical alterations that contribute to the clinical presentation of BPD.

### 1.2. Current Limitations

Despite the growing body of neurobiological research, which includes evidence of reduced prefrontal cortex activity, heightened amygdala reactivity, hippocampal volume loss, neurotransmitter dysregulation, and genetic/epigenetic alterations, there is still a significant gap between research findings and their application in clinical practice. These findings are rarely incorporated into routine diagnostic evaluations or therapeutic decision-making processes. Current treatment approaches continue to rely largely on psychotherapeutic interventions, with little stratification of patients or symptom clusters based on biological factors.

Furthermore, although some promising tools have emerged (e.g., neuroimaging biomarkers, neuromodulation, and pharmacogenetics), ethical concerns, technological constraints, and a lack of consensus regarding their practical utility mean they are underutilized in clinical practice. This results in a missed opportunity to apply the principles of precision psychiatry to a population that urgently needs effective, individualized care.

### 1.3. Rationale for This Review

Due to the increasing prevalence of BPD, particularly among adolescents, and its associated socioeconomic impact, there is an increasing demand for the integration of neurobiological insights into diagnostic, therapeutic, and preventive strategies. Therefore, an up-to-date, comprehensive, and interdisciplinary synthesis is essential in order to achieve the following:

- Clarify the current understanding of BPD’s neurobiological mechanisms;
- Highlight translational gaps that hinder clinical application;
- Inform the development of personalized and multimodal treatment approaches.

This review addresses this need by systematically organizing recent findings from neuroscience, genetics, and psychiatry, and by interpreting their relevance through a clinically oriented lens.

### 1.4. Objectives

The primary objectives of this review are as follows:

- To synthesize current evidence on the structural, functional, and neurochemical brain changes associated with BPD;
- To explore the role of genetic and epigenetic mechanisms, particularly in relation to early trauma;
- To discuss the cognitive and developmental implications of neurobiological alterations;
- To identify how neurobiological insights can inform clinical practice, including diagnosis, risk assessment, and treatment.
- To highlight future research directions, particularly those focused on biomarker validation, therapeutic innovation, and the development of integrated care models.

This review is organized around four core questions designed to assess the validity, consistency, and translational value of neurobiological findings in BPD, to guide the narrative synthesis. These core questions are as follows:

(1) Which structural, functional, or neurochemical abnormalities remain significant after controlling for key comorbidities, such as depression, PTSD, and ADHD?
(2) Which neuroimaging techniques demonstrate sufficient test–retest reliability (≥0.6) and replicability across studies?
(3) To what extent do neurobiological markers differentiate BPD from other personality and affective disorders (i.e., transdiagnostic specificity)?
(4) Which biomarkers show promise as clinically relevant predictors of treatment response or symptom trajectories?

These guiding questions serve as an analytic framework throughout the Results and Discussion sections, highlighting the most robust and clinically meaningful patterns in the literature.

## 2. Methodology

This article provides a systematic narrative review of the existing literature on the neurobiological basis of borderline personality disorder (BPD). Rather than presenting original empirical data, it synthesizes and critically evaluates findings from multiple disciplines, including neuroscience, genetics, neuropsychology, and psychiatry, in order to bridge the gap between current research and clinical application.

### 2.1. Review Type and Justification

We employed a systematic narrative review design, which is particularly well-suited to complex and heterogeneous fields, such as BPD research. The diversity of methods, outcome measures, and populations in this field makes quantitative meta-analysis less feasible. This approach enables the thematic integration of findings, the conceptual comparison of results across different areas of research, and an interpretative depth that goes beyond the mere aggregation of effect sizes.

### 2.2. Search Strategy

A structured and comprehensive search strategy was implemented to identify the relevant literature on the neurobiological mechanisms underlying borderline personality disorder. The search was conducted across three major scientific databases, namely PubMed, PsycINFO, and Scopus. This covered publications from 1 January 2000 to 31 May 2025. The 25-year timeframe was chosen to encompass both foundational neurobiological research and the most recent advancements, particularly in neuroimaging, genetics, and epigenetics.

To ensure comprehensive coverage, the search was expanded to include the Web of Science Core Collection. The final Boolean search string was as follows:

(“borderline personality disorder” OR “BPD”) AND (“neuroimaging” OR “fMRI” OR “MRI” OR “functional connectivity” OR “neurobiology” OR “brain structure” OR “neurotransmitters”).

**Figure 1:**
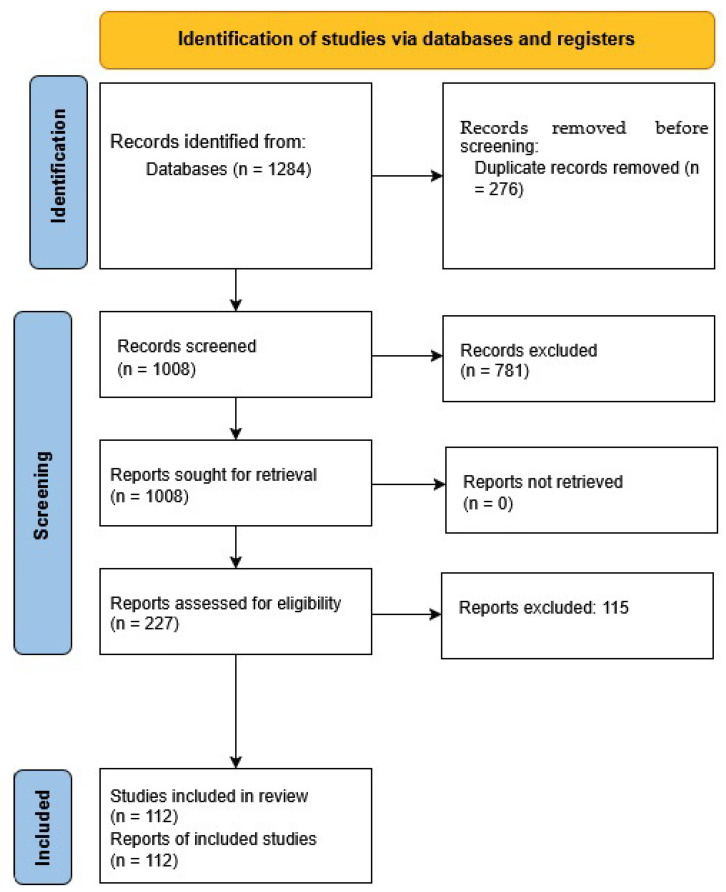
PRISMA flow diagram of the study selection process [10]. A total of 1284 records were identified through database searches. After removing 276 duplicates, 1008 records were screened by title and abstract. Of these, 781 were excluded, and 227 full-text articles were assessed for eligibility. Following the exclusion of 115 full-text articles, 112 studies were included in the final synthesis.

Searches were limited to English-language peer-reviewed articles published between 2000 and 2025. Truncation operators (e.g., neuro) and Boolean logic were adapted for the syntax of each database. The number of records retrieved from each database is reported in the PRISMA flow diagram (Figure 1).

### 2.3. Search Terms and Syntax

The search terms were constructed using Boolean operators and comprised both controlled vocabulary (e.g., MeSH terms) and free-text keywords. The following combinations were used:

“borderline personality disorder” AND (“neurobiology” OR “brain structure” OR “brain function”).

“BPD” AND (“neuroimaging” OR “fMRI” OR “PET” OR “functional connectivity”).

“borderline personality disorder” AND (“genetics” OR “epigenetics” OR “gene-environment interaction”).

“BPD” AND (“neurotransmitters” OR “serotonin” OR “dopamine” OR “glutamate”).

“borderline personality disorder” AND “cognitive deficits” OR “executive function” OR “working memory”.

The search was restricted to peer-reviewed journal articles published in English. Where available, filters were applied to narrow the results to “human studies” and “adolescents/adults”.

### 2.4. Inclusion Criteria

- Studies published between 2000 and 2025.
- Articles focusing on individuals with a clinically diagnosed BPD based on DSM 5 or ICD-11 criteria.
- Studies must report empirical data (e.g., neuroimaging, genetics, epigenetics, neurochemistry, or cognition).
- Meta-analyses and systematic reviews relevant to the neurobiology of BPD.
- Studies examining comorbidity or transdiagnostic features were only included if they provided BPD-specific data.

### 2.5. Exclusion Criteria

- Non-empirical publications (e.g., theoretical articles, letters, commentaries and editorials).
- Case reports or single-subject studies lacking generalizability.
- Animal or preclinical studies not involving human subjects.
- Articles that were not available in full text or that were published in a language other than English.
- Studies where BPD was a secondary or incidental focus without subgroup analysis.

### 2.6. Database Filtering and Screening Process

The initial results were exported to reference management software (e.g., Zotero or EndNote), and duplicate entries were removed. Two independent reviewers then screened all titles and abstracts to assess their relevance. The full texts of articles that met the inclusion criteria or where the relevance could not be determined from the abstract alone were retrieved.

Any disagreements regarding inclusion were resolved through consensus discussion, with a third reviewer available for arbitration if necessary.

### 2.7. Selection and Categorization

All retrieved articles were screened for relevance using predefined inclusion and exclusion criteria. Studies with fewer than 20 participants per group were excluded, unless this was justified by a replication or mechanistic design. This threshold corresponds to a power estimate of 0.8 for detecting a medium effect size (*d* = 0.5) at α = 0.05. The selected studies were then categorized thematically into the following five domains:

- Brain structure and function.
- Neurotransmitter systems.
- Genetic and epigenetic factors.
- Developmental contributions (e.g., childhood trauma).
- Cognitive and functional connectivity.

The study selection process is summarized in Figure 1 using a PRISMA-style flow diagram, which shows how many studies were included or excluded at each stage of the review.

### 2.8. Data Synthesis

A narrative synthesis approach was taken to qualitatively analyze the findings and identify converging and diverging trends. Rather than aggregating effect sizes, the review emphasizes the integration of theory and contextual understanding of neurobiological mechanisms across studies. This approach takes into account disciplinary diversity, heterogeneous methodologies, and the complexity of BPD’s symptomatology.

### 2.9. Quality Assessment and Statistical Considerations

As this is a narrative synthesis, no original data collection or statistical analysis was conducted by the authors. Consequently, references to statistical methods (e.g., *t*-tests, ANOVA, effect sizes) reflect the methodologies and results reported in the included studies and are cited accordingly. No statistical software was used, nor were new thresholds for significance defined within this manuscript.

Included studies were selected based on their use of validated neuroimaging protocols (e.g., fMRI, PET, DTI with standardised pre-processing pipelines) and psychometric instruments with established reliability (e.g., structured clinical interviews, cognitive batteries, symptom rating scales), to ensure methodological rigor and interpretive reliability, Studies that clearly described diagnostic criteria (e.g., DSM-IV/5, ICD-10/11), inclusion and exclusion procedures, and applied robust quality control measures were given preference.

Each included study was also independently evaluated using the Joanna Briggs Institute (JBI) Checklist for Analytical Cross-Sectional Studies, adapted for neuroimaging research. Two reviewers conducted the assessments independently, and inter-rater reliability was calculated using Cohen’s kappa (κ = 0.82), indicating substantial agreement between raters. The resulting quality scores are tabulated in Supplementary Table S1. Studies scoring below 70% were excluded from the synthesis to ensure the reliability of the interpretation. Finally, sample size adequacy was considered during the quality appraisal: studies with fewer than 20 participants per group were excluded.

## 3. Functional Connectivity Disruptions in Borderline Personality Disorder

### 3.1. Prefrontal–Amygdala Dysconnectivity

Disruptions in communication between the prefrontal cortex and limbic structures, particularly the amygdala, have been strongly linked to deficits in emotional regulation and social cognition. This dysconnectivity contributes to impulsivity, emotional instability, and heightened threat perception, which are core symptoms in BPD. These disruptions limit the modulation of emotional responses from higher-order brain regions and are frequently associated with executive dysfunction and memory impairment.

### 3.2. Default Mode Network Abnormalities

Dysfunction of the default mode network (DMN), particularly in the medial prefrontal cortex (mPFC) and posterior cingulate cortex (PCC), is consistently observed in individuals with BPD. These regions play a key role in self-referential processing, autobiographical memory, and emotional regulation. Abnormal DMN activity has been linked to a fragmented self-concept and persistent negative rumination [11,12], which can exacerbate emotional instability and identity disturbances associated with BPD.

### 3.3. Social Brain and Mentalization Networks

Dysfunctions in the mentalizing network and the mirror neuron system impair the ability to understand and reflect on others’ mental states. These deficits impair empathy, increase hypersensitivity to rejection, and lead to the misinterpretation of social cues. This makes interpersonal interactions more aversive and unstable [13,14].

### 3.4. Stress-Response System (HPA Axis) and Dynamic Reactivity

Hyperactivation of the hypothalamic–pituitary–adrenal (HPA) axis is a hallmark of BPD, reflecting increased stress reactivity. Early trauma exposure contributes to long-term HPA axis dysregulation via epigenetic modifications, thereby heightening emotional lability and physiological stress sensitivity [15,16]. These changes interact dynamically with neural networks governing threat appraisal and emotion regulation.

### 3.5. Mirror Neuron System and Interpersonal Misattunement

Altered functioning of the mirror neuron system in BPD further disrupts interpersonal attunement and emotional resonance. These abnormalities impair the intuitive understanding of others’ intentions and emotional states, leading to increased social withdrawal, heightened sensitivity to perceived rejection, and interpersonal dysfunction [13,14].

### 3.6. Machine Learning and Graph Theoretical Approaches

Recent advances in multiscale connectomics and computational psychiatry have begun to transform our understanding of BPD as a disorder involving the organization of dynamic brain networks. Using supervised machine learning classifiers and graph theoretical modelling, new findings have revealed specific neurotopological signatures that can distinguish individuals with BPD from healthy controls.

A 2025 study employing multiscale support vector machines (SVM) and elastic net regularization achieved a mean classification accuracy of 71% (precision = 0.73; recall = 0.68), based on a feature space comprising both static and time-varying functional connectivity patterns [17]. The most discriminative regions included the precuneus, superior temporal sulcus, dorsal anterior cingulate cortex (dACC), and amygdala–default mode network (DMN) interactions, supporting the notion of altered integration within self-referential and social-cognitive networks.

Graph theoretical analysis also revealed reduced global efficiency and mean nodal degree, particularly in the middle temporal gyrus, insula, and orbitofrontal cortex. Notably, these neural features were significantly correlated with maladaptive personality traits measured via the PID-5, including separation insecurity and depressivity. This suggests a possible neurobiological substrate for affective instability and interpersonal dysfunction in BPD.

These results provide preliminary, yet encouraging, support for the use of machine learning models and network-based biomarkers as diagnostic tools and for categorizing patient subgroups based on personality-linked neural signatures. Future research should focus on cross-validation in larger cohorts and integration with longitudinal treatment outcome data.

## 4. Neurostructural and Network-Level Correlates

### 4.1. Prefrontal Cortex

The prefrontal cortex (PFC), particularly the dorsolateral and ventromedial regions, plays a crucial part in controlling impulses, making decisions, and regulating emotions. Consistent findings from structural MRI studies report reduced grey matter volume in these regions in individuals with BPD [18], which suggests compromised top-down control of limbic hyperactivity. These findings support clinical observations of impaired judgment and emotional instability.

### 4.2. Amygdala

The amygdala, which is central to the processing of emotional salience, often shows increased volume or heightened activation in patients with BPD. This hyperresponsiveness is associated with exaggerated reactions to perceived threats or social rejection. Structural variability in amygdala subnuclei has been linked to impulsive aggression and emotional dysregulation, which is functional evidence of fronto–limbic dysconnectivity [19].

### 4.3. Hippocampus

Numerous neuroimaging studies on BPD have documented reduced hippocampal volume, particularly among individuals with a history of early trauma or chronic stress. Given the role of the hippocampus in contextual memory and the regulation of the hypothalamic–pituitary–adrenal (HPA) axis, structural compromise in this region may underlie intrusive memories, identity fragmentation, and emotional hyperarousal [20,21].

### 4.4. Parietal Cortex

**Figure 2:**
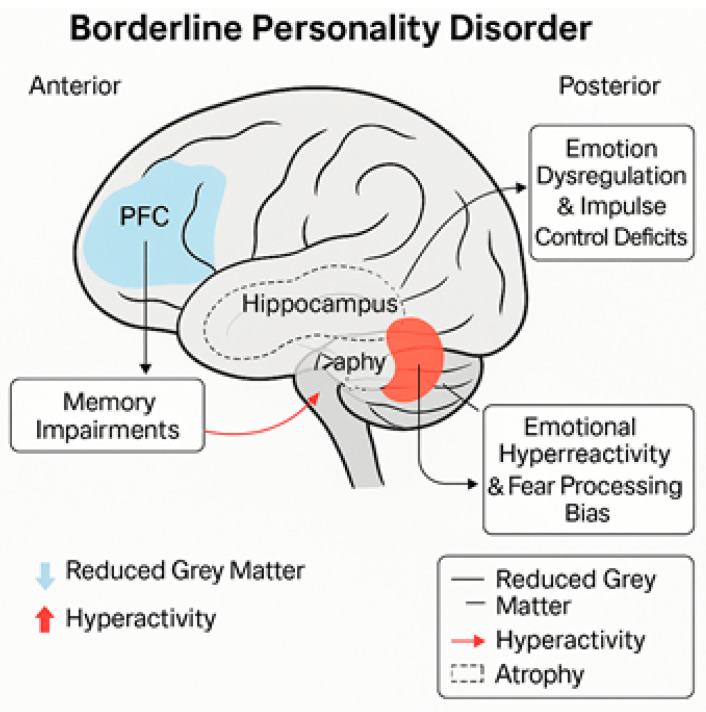
Structural and functional brain alterations in borderline personality disorder. Schematic representation of key neuroanatomical abnormalities in BPD and their associated functional deficits.

There is growing evidence that abnormalities in the parietal cortex are responsible for the visuospatial and constructional deficits in BPD. Abnormal parietal activity has been associated with problems in planning and organizing space, leading to deficits in executive function [22]. Figure 2 portrays the key neuroanatomical abnormalities in BPD and their associated functional deficits.

### 4.5. Personality Dimensions and Network Efficiency

There is emerging evidence that highlights the role of personality traits as network-level endophenotypes in BPD. A 2025 connectomic study [17] linked specific PID-5 domains, including separation insecurity, depressivity, and emotional lability, to alterations in large-scale brain network organization. Individuals with higher scores on these maladaptive traits exhibited significantly reduced global network efficiency and decreased nodal degree in key socio-emotional hubs, including the middle temporal gyrus, insula, and posterior cingulate cortex.

Notably, these disruptions were observed not only at the static connectivity level, but also during dynamic transitions between network states. High depressivity scores were associated with prolonged dwell time in hypo-connected default mode states, while separation insecurity predicted reduced integration across salience and executive control networks. This suggests impaired affect regulation and goal-directed adaptation.

These findings support the idea that dimensional personality features, as measured by instruments, such as the PID-5, may serve as transdiagnostic markers of vulnerability across affective and personality spectrum disorders. Therefore, integrating personality trait measures with graph theoretical models holds promise for more individualized diagnosis and treatment planning in BPD and related psychopathologies.

### 4.6. Meta-Analytic Findings

Recent meta-analyses have replicated these neuroanatomical deficits and their functional counterparts.

- Hyperactivation of the amygdala is one such deficit. This has been replicated consistently across studies, particularly in tasks involving emotion stimuli [23,24].
- Hypoactivation of the prefrontal cortex (PFC): Decreased activation in the DLPFC and ACC disrupts the integration of cognitive and emotional processing, leading to heightened impulsivity and emotional instability [25,26].
- Hippocampal volume reduction: This is linked to spatial memory deficits and increased emotional reactivity [19,21,27].

Together, these neuroanatomical findings highlight the extent to which structural deficits and connectivity disruptions are responsible for the clinical symptoms of BPD. For instance, amygdala hyperactivation and prefrontal cortex (PFC) hypoactivation provide a neurobiological basis for emotional hypersensitivity and impaired impulse control. Additionally, hippocampal volume loss explains the emotional and cognitive dysregulation characteristic of the disorder.

## 5. Neurobiological Underpinnings

The neurobiological basis of BPD involves a synergistic combination of neurotransmitter dysfunction, pathological brain network connectivity, and dysregulation of the stress regulatory systems (Table 2). These elements together are major contributors to the characteristic manifestations of emotional dysregulation, impulsivity, and problematic interpersonal relationships.

### 5.1. Neurotransmitter Dysfunctions

**Figure 3:**
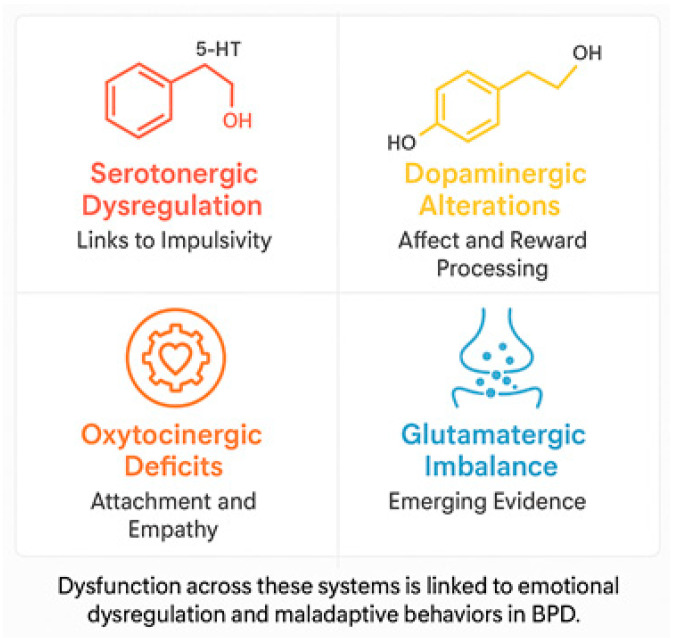
Neurochemical systems implicated in BPD. Schematic representation of disrupted neural network connectivity associated with core symptoms of BPD. Highlights include impaired top-down regulation from the prefrontal cortex to the amygdala, dysregulated default mode network activity, and deficits in social cognition networks. These disruptions contribute to affective instability, identity disturbance, and interpersonal dysfunction.

Various neurotransmitter systems are implicated in the pathophysiology of BPD (Figure 3), as follows:

- Serotonin (5-HT): Dysregulation of the serotonergic system has been associated with the impulsive behavior, aggression, and mood swings that are typical of BPD. Reduced serotonin activity, particularly in the prefrontal cortex (PFC), is believed to be associated with difficulty controlling impulses and increased aggression [33,34]. Selective serotonin reuptake inhibitors (SSRIs) have demonstrated some therapeutic benefit in managing these symptoms, though the response may vary significantly between individuals [35].
- Dopamine (DA): Dysfunctions in the dopaminergic system have been associated with disturbances in reward processing, emotional regulation, and impulsivity. Neuroimaging studies have revealed alterations in mesolimbic circuitry and decreased dopamine receptor binding in the striatum, which are linked to heightened emotional sensitivity and impaired regulation of negative emotions [36,37].
- Glutamate: Recent evidence suggests that alterations in the glutamatergic system are associated with borderline personality disorder. Dysregulation of excitatory neurotransmission can exacerbate emotional instability and reinforce impulsive decision-making [11].
- Oxytocin and the opioid system: Disturbance of oxytocin, a neuropeptide that plays an essential role in attachment mechanisms and social cognition, is associated with the relational difficulties observed in BPD. Decreased oxytocin levels may impair the processing of emotional information, thereby exacerbating interpersonal relationship problems [32,38]. Furthermore, dysregulation in the endogenous opioid system is linked with chronic dysphoria and self-injurious behavior, which temporarily alleviates emotional distress by increasing opioid levels [31].

### 5.2. Functional Connectivity in Borderline Personality Disorder

**Figure 4:**
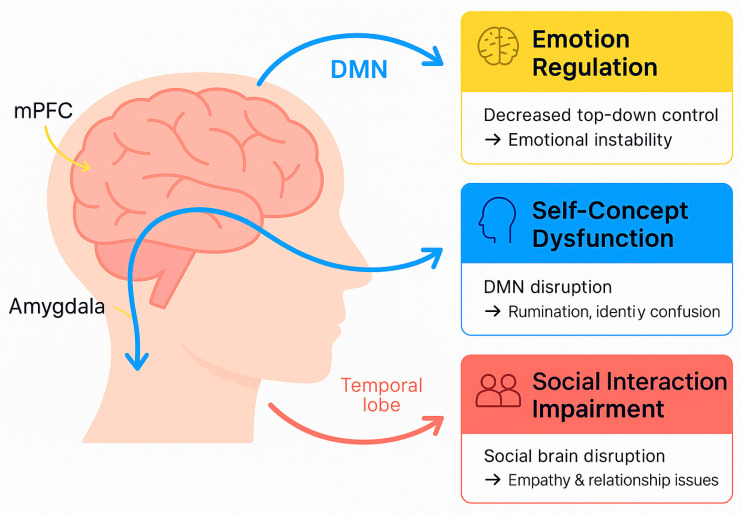
Functional connectivity disruptions in borderline personality disorder (BPD). Summary diagram of key neurotransmitter systems involved in BPD pathophysiology. Includes serotonergic dysregulation (linked to impulsivity), dopaminergic alterations (related to reward processing and affect), oxytocinergic deficits (associated with attachment and empathy), and emerging evidence on glutamatergic imbalance.

Disruptions to functional connectivity within the key brain networks involved in emotion regulation and social cognition are central to our understanding of the neurobiological foundations of BPD. These abnormalities are the basis of disorder’s defining features, such as emotional dysregulation, impulsivity, and interpersonal difficulties (Figure 4).

### 5.3. Key Connectivity Disruptions

- Prefrontal Cortex (PFC) and Amygdala Connectivity:

A decrease in top-down regulatory control from the prefrontal cortex (PFC) to the amygdala enhances emotional reactivity, particularly in response to fear and anger. This impaired regulation can lead to impulsivity and emotional instability. One study demonstrated that these disruptions result in an inability to regulate emotional responses and stress, which is characteristic of BPD [30].

- Default mode network (DMN) dysfunction:

Deficits in the DMN, primarily in the medial prefrontal cortex (mPFC) and posterior cingulate cortex (PCC), interfere with self-referential thought processes. This decline indicates a breakdown of the self-concept and relentless negative ruminative thinking. Abnormalities in the DMN, which is involved in self-referential thinking and emotional regulation, have been observed in patients with BPD [11]. One study examined the relationship between DMN dysfunction and impaired self-concept and negative rumination, which reinforces emotional lability [12].

- Social brain networks dysfunction:

Dysfunctions in the mentalizing network and mirror neuron system disrupt the ability to empathize with the feelings and intentions of others, heighten sensitivity to rejection cues, and render social interactions more aversive. Contemporary studies demonstrated that these dysfunctions exacerbate one of the significant challenges for people with BPD, namely poor interpersonal relationships [13,14].

### 5.4. Stress-Response System Dysregulation

Dysregulation of the hypothalamic–pituitary–adrenal (HPA) axis, which plays a central role in the stress response system, is a defining characteristic of BPD. Secondary hypercortisolism resulting from HPA axis overactivation increases stress sensitivity and emotional reactivity. Exposure to chronic early trauma has been found to cause epigenetic changes in HPA axis functioning, making individuals more vulnerable to emotional lability [15,16].

### 5.5. Electrophysiological Biomarkers

Event-related potentials (ERPs), specifically the P300 component, are regarded as potential biomarkers of BPD. Variability in P300 amplitude indicates abnormal processing of emotional information and may reflect the neurophysiological basis of symptoms of trauma-related symptoms [39,40].

These findings emphasize the complex neurobiological mechanisms underlying BPD and highlight the necessity of an integrated treatment approach. More specifically, interventions targeting the serotonergic and dopaminergic systems, such as selective serotonin reuptake inhibitors (SSRIs) and antipsychotics, show promise, but require customization to address the disorder’s heterogeneity. Similarly, neuromodulation techniques, such as transcranial magnetic stimulation (TMS), show potential in addressing deficits in functional connectivity, particularly between the prefrontal cortex (PFC) and the amygdala. Table 2 provides a comparative summary of key neurobiological findings across domains implicated in BPD.

## 6. Genetic and Epigenetic Factors

The pathophysiology of BPD is an interactive process involving environmental factors and genetic predisposition. Although genetic factors play a significant role in the development of the disorder, epigenetic changes, particularly those resulting from early-life stress, provide valuable insight into how environmental stressors influence the neurobiological basis of BPD.

### 6.1. Genetic Contributions

Family, twin, and adoption studies consistently indicate that BPD traits are heritable, with an estimated heritability coefficient of around 46% [41,42]. Twin studies further demonstrate that monozygotic twins have an 11.5 times higher risk of developing BPD than dizygotic twins, emphasizing the disorder’s strong genetic component. However, the specific genetic underpinnings remain largely unknown.

Most genetic studies of BPD have focused on candidate genes within the serotonergic, dopaminergic, and noradrenergic systems, but the results have been inconsistent. Notably, genetic polymorphisms in the serotonin transporter gene (5-HTTLPR) have been associated with increased emotional reactivity and impulsivity [33]. Similarly, variations in the dopamine receptor D4 gene (DRD4) have been linked to impulsive behavior and novelty-seeking tendencies, which are commonly observed in BPD [43,44].

Further research highlights substantial genetic overlap between BPD and other psychiatric disorders, including bipolar disorder, major depressive disorder, and schizophrenia, suggesting shared genetic risk factors [45]. Despite these insights, large-scale genome-wide association studies (GWAS) remain scarce. To date, the only GWAS study of BPD included fewer than 1000 subjects and failed to identify any variants that were significant across the whole genome.

Genetic research is crucial for identifying new treatment targets and developing preventive strategies for BPD. Further research is needed.

### 6.2. Epigenetic Contributions

Epigenetic changes, and more particularly DNA methylation, explain how environmental risk factors, such as early childhood trauma, influence gene expression and neurodevelopment.

- Early-life trauma effect: Adverse events, including abuse and neglect, during early years can leave epigenetic marks that disrupt stress regulation mechanisms. For example, hypermethylation of the NR3C1 gene, which encodes the glucocorticoid receptor, has been observed in patients with borderline personality disorder, suggesting a potential disruption in the hypothalamic–pituitary–adrenal (HPA) axis [15,16]. This dysregulation increases the vulnerability to emotional dysregulation and hyperactive stress responses.
- The interplay between genetic predisposition and environmental stress conditions is a fundamental component to BPD pathogenesis. For instance, individuals with certain polymorphisms of the 5-HTTLPR gene are more susceptible to traumatic events during their early years and exhibit heightened emotional and behavioral responses [46,47].

### 6.3. Genetic and Epigenetic Studies’ Limitations

- Lack of specificity: Although candidate genetic and epigenetic markers have been suggested, no gene or epigenetic alteration has been reliably linked to BPD. The disorder’s heterogeneity and high comorbidity levels further complicate the identification of definitive markers.
- Limited sample sizes: Many genetic and epigenetic BPD-specific investigations have small sample sizes, which makes their findings less generalizable and credible [9].
- Transdiagnostic factors: The majority of the genetic and epigenetic changes that were observed are not specific to BPD, but appear in various other psychiatric disorders, including PTSD, depression, and anxiety, and are, therefore, not viable as specific biomarkers [48].

Genetic and epigenetic research implies that early interventions are necessary to avert the impact of environmental stressors on vulnerable individuals. For example, intervening in stress-regulation pathways and enhancing resilience through psychosocial interventions could reduce the long-term risk of developing BPD. Furthermore, understanding gene–environment interactions could lead to the development of personalized treatments, such as pharmacogenetic interventions based on the patient’s genetic profile.

## 7. Neurodevelopmental Contributions

BPD is subject to powerful neurodevelopmental influences, with early experience playing a key role in determining the disorder’s trajectory. Childhood adversity, attachment disruption, and trauma can have a profound impact on the developing brain, inducing structural and functional changes that underpin the emotional and interpersonal difficulties characteristic of BPD.

### 7.1. Effect of Childhood Adversity

Adverse childhood experiences (ACEs), including physical, emotional, and sexual abuse, as well as neglect, are strongly associated with the development of BPD. Over 70% of patients with BPD have reported severe trauma in early life, which is one of the most consistent environmental risk factors for the disorder [47,49].

- Structural and functional consequences: Repeated exposure to trauma during critical periods of neurodevelopment can interfere with the normal maturation of key brain structures, such as the hippocampus, amygdala, and prefrontal cortex [21,46]. These structures play a significant role in regulating emotions, consolidating memories, and adapting to stress.
- HPA axis dysregulation: Early-life adversity is associated with hyperactivation of the hypothalamic–pituitary–adrenal (HPA) axis, resulting in increased cortisol levels and heightened stress sensitivity [15,16] dysregulation is responsible for the emotional dysregulation and impulsive behavior exhibited in BPD.

### 7.2. Attachment Disruptions

The attachment theory framework provides valuable insight into the interpersonal issues associated with BPD. Patients with BPD typically exhibit insecure and disorganized patterns of attachment, often stemming from inconsistent or neglectful caregiving behaviors [50].

- Neurodevelopment: Disruptions to early attachment relationships can adversely affect the limbic and cortical systems, emotional regulation, and social cognition. The amygdala becomes hyperactive, and the prefrontal cortex less connected, thereby enhancing interpersonal hypersensitivity and problems with emotional regulation [32,51].
- Impairments in mentalization: Insecure attachment disrupts the development of mentalization, or the ability to understand others’ mental states. Impaired mentalization is a central feature of BPD, leading to misunderstandings of emotional cues and hypersensitivity to rejection [52].

### 7.3. Developmental Timing and Vulnerability

The temporal characteristics of traumatic experiences and adversity are critical to assessing their influence on neurodevelopment. Traumatic experiences in early childhood, when maximal brain plasticity is present, have the greatest impact on both brain structure and potential functioning [51,53].

- Critical periods: Early windows of development are crucial in forming neural pathways related to the stress response and emotional regulation. Disruption during these periods can result in permanent deficits in self-regulation, attachment, and cognitive processing [46].
- Social contexts: Chronic social stressors, such as bullying, social deprivation, or re-victimization, also contribute to developmental vulnerabilities, perpetuating the cycle of emotional dysregulation and interpersonal conflict in BPD [54].

### 7.4. Neurodevelopmental Mechanisms

- Neuroplasticity: Although adverse experiences in early life can have a negative effect on brain development, the concept of neuroplasticity offers a positive outlook on the potential for intervention. Some therapeutic interventions, such as dialectical behavior therapy (DBT) and mentalization-based therapy (MBT), can restructure maladaptive neural circuits and achieve functional adaptation [53].
- Epigenetics: Epigenetic alterations provide a mechanistic link between early experience and subsequent neurodevelopmental outcomes. For example, methylation of the glucocorticoid receptor gene (NR3C1), which is linked to trauma, affects the functioning of HPA, resulting in heightened emotional reactivity and stress sensitivity [15].

The neurodevelopmental model of BPD emphasizes the importance of early intervention. Preventive interventions, such as parental competency strengthening, developing secure attachment relationships, and providing trauma-informed care can potentially avert the chronic neurobiological impact of childhood trauma. Furthermore, treatments utilizing mentalization and emotional regulation have the capacity to reorganize aberrant neural networks and improve outcomes in BPD.

## 8. Cognitive Deficits

BPD is also associated with significant cognitive deficits spanning numerous domains, such as executive function, memory, attention, and visuospatial skills. These deficits account for many of the difficulties experienced by individuals with BPD in their daily lives and during treatment.

### 8.1. Executive Functioning

Executive dysfunction is a defining feature of BPD, manifesting as difficulties with planning, impulse control, problem-solving, and cognitive flexibility.

- Deficits: Meta-analyses suggest that subdomain of executive functioning most affected by BPD is inhibition. This is characterized by an inability to inhibit impulsive responses and manage emotions [22]. These deficits in inhibition subsequently hinder decision-making processes and flexibility in complex situations.
- Neuroanatomical correlates: Impairment of the dorsolateral prefrontal cortex (DLPFC), which plays a key role in working memory, goal-oriented behavior, and self-regulation, leads to cognitive impairment [55,56].

### 8.2. Memory Impairments

Memory impairments in BPD are specific to certain domains and directly impact long-term spatial memory.

- Spatial vs. verbal memory: Long-term spatial memory is severely impaired, whereas long-term verbal memory remains largely intact. This may be due to differential levels of hippocampal activation, with the hippocampus being more active during the processing of spatial information [22].
- Emotional memory: Affect dysregulation in BPD can exacerbate memory distortions, particularly for emotionally significant or traumatic experiences. There is impaired memory for neutral events, but increased memory for negative emotional stimuli [57].

### 8.3. Attention and Visuospatial Skills

Individuals with BPD also exhibit attentional deficits and impairments of visuospatial skills.

- Attention: Deficits in sustained and selective attention are a hallmark of BPD, causing difficulties in work, school, and interpersonal relationships [58].
- Visuospatial skills: Deficits in spatial planning and organization, linked to parietal cortex pathology, disrupt the ability to navigate through complex tasks involving constructional and visual processing [22].

### 8.4. Functional Connectivity and Cognitive Deficits

Dysregulated connectivity between the prefrontal cortex (PFC) and limbic structures, such as the amygdala and hippocampus, interferes with the integration of emotional and cognitive processes in BPD [59]. This disruption worsens deficits in emotion regulation during decision making and prevents effective cognitive control under high levels of stress.

BPD-related cognitive impairment significantly influences both treatment outcomes and everyday functioning. Treatment through targeted therapeutic interventions, such as cognitive remediation therapy or mindfulness-based interventions, can enhance cognitive flexibility, emotional regulation, and overall adaptive functioning. Furthermore, identifying particular cognitive profiles in individuals with BPD can inform personalized treatment planning and enhance the therapeutic alliance.

## 9. Clinical Implications

Significant progress has been made in understanding the neurobiology and psychological mechanisms of BPD, which has major implications for improving diagnosis, treatment, and prevention. These emerging findings highlight the importance of integrating biological, psychological, and social models in order to develop personalized and effective treatment strategies (Table 3).

### 9.1. Improved Diagnostic Accuracy

- Biomarkers: Neuroimaging techniques have revealed changes in the morphology of key brain regions, particularly the prefrontal cortex (PFC), amygdala, and hippocampus, which play a crucial role in emotion regulation and impulsive behavior.
- Electrophysiological markers: The P300 component has been identified as an effective biomarkers for BPD, offering promising avenues for enhancing diagnostic precision and monitoring therapeutic response [39,40].
- Heterogeneity and comorbidities: Distinguishing BPD from other psychiatric disorders, including PTSD, bipolar disorder, and major depression, will be important in clarifying BPD heterogeneity. Combining neurobiological data with clinical evaluation could clarify the characteristics that are shared versus those that are specific to each disorder [9].

### 9.2. Therapeutic Interventions

Psychotherapy: Dialectical behavior therapy (DBT) and mentalization-based therapy (MBT) are the two psychotherapeutic approaches with the most empirically supported for BPD, and there is growing evidence of their neurobiological impact. DBT, developed by Linehan, emphasizes acquiring emotional regulation, distress tolerance, mindfulness, and interpersonal skills. Neuroimaging findings suggest that DBT modulates fronto–limbic circuitry by enhancing prefrontal cortical regulation and reducing hyperactivity in the amygdala, thereby improving impulse control and emotional reactivity [26,60].

MBT, introduced by Bateman and Fonagy is rooted in attachment theory and aims to enhance reflective functioning and social cognition by restoring mentalizing capacities. It engages brain regions, such as the medial prefrontal cortex and the temporoparietal junction, both of which are often impaired in individuals with BPD. Improved mentalization is associated with increased stability of the self-concept and reduced interpersonal dysregulation [61,62]. Together, these therapies target the psychological and neurobiological dimensions of BPD, supporting their use in integrated treatment models.

Alongside DBT and MBT, which have demonstrated both clinical and neurobiological efficacy [26,60,61,62], emerging approaches, such as cognitive remediation therapy and mindfulness-based interventions, show promise in enhancing cognitive flexibility, emotional regulation, and daily functioning in individuals with BPD [22].

Cognitive remediation therapy (CRT) addresses specific cognitive deficits often seen in BPD, such as impaired working memory, planning, and response inhibition. By employing repetitive and adaptive tasks, CRT promotes neuroplastic changes in the frontoparietal circuits involved in executive function, leading to improvements in cognitive flexibility and self-regulation [22,63].

Mindfulness-based interventions (MBIs), such as mindfulness-based cognitive therapy (MBCT) and mindfulness-based stress reduction (MBSR), focus on enhancing attentional control, present-moment awareness, and emotion regulation. These approaches have been shown to reduce amygdala hyperactivation and increase prefrontal cortical engagement during emotional tasks, thereby modulating the neural circuitry underlying affective dysregulation in BPD [60,64]. MBIs may also reduce dissociative symptoms and ruminative thinking, which are common in individuals with high emotional reactivity.

These therapies may complement established models by addressing the deficits in executive function and attentional control deficits identified in neuropsychological studies. Regarding pharmacological treatments, SSRIs and mood stabilizers have been found to be ineffective in managing mood lability and impulsivity, particularly in cases of dysregulations in the serotonergic and dopaminergic systems [30,34].

- Novel approaches to tailoring pharmacotherapy to genetic polymorphisms, e.g., serotonin transporter and dopamine receptor gene polymorphisms, to realize optimal therapeutic gains [65].
- Neuromodulation: Approaches, such as transcranial magnetic stimulation (TMS) and transcranial direct current stimulation (tDCS), offer promising non-invasive interventions for BPD. TMS uses magnetic pulses to stimulate specific brain regions, most commonly the dorsolateral prefrontal cortex (DLPFC), which is involved in executive functioning and emotion regulation. TMS has been shown to increase prefrontal activity and reduce limbic hyperreactivity, which could improve emotional control and impulsivity in patients with BPD [66,67].

By contrast, tDCS applies a low-intensity electrical current to modulate cortical excitability. Anodal stimulation over the DLPFC can enhance cognitive control, while cathodal stimulation over limbic areas may reduce emotional overactivation. While research on tDCS in BPD remains limited, initial findings suggest that it could enhance attentional focus and reduce emotional lability [68]. These interventions are safe and well-tolerated and have the potential to be used as adjunctive treatments targeting core neurobiological dysfunctions in BPD in the future.

- Longitudinal effects of psychodynamic psychotherapy on neural activity and emotional functioning:

Emerging longitudinal evidence suggests that psychodynamic psychotherapy exerts measurable effects on brain function and emotional regulation in individuals with bipolar disorder (BP). A recent 2024 neuroimaging study conducted over 12 months found that weekly psychodynamic sessions were associated with a progressive normalization of dorsal anterior cingulate cortex (dACC) activation, particularly in response to affective stimuli and conflict monitoring tasks [69]. These neural changes were accompanied by a gradual reduction in alexithymia, as measured by the Toronto Alexithymia Scale (TAS-20), indicating an improvement in emotional awareness and regulation over time.

**Figure 5:**
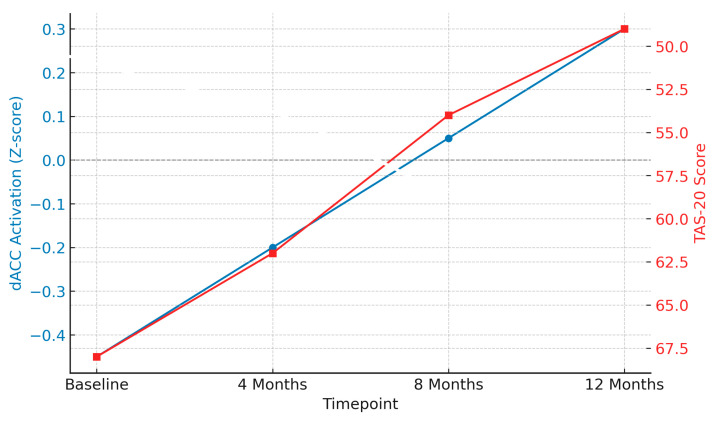
Longitudinal change in dorsal anterior cingulate cortex (dACC) activation and alexithymia (TAS-20 scores) over 12 months of psychodynamic psychotherapy in individuals with borderline personality disorder (BPD). dACC activation (blue line) shows progressive normalization (increasing Z-scores), while TAS-20 scores (red line) decrease over time, indicating improved emotional awareness. Measurements were taken at baseline, 4 months, 8 months, and 12 months. Lower TAS-20 scores reflect therapeutic progress. Data adapted from [69].

Figure 5 illustrates the temporal trajectory of these effects, showing comparative mean z-scores in dACC activation and TAS-20 scores at baseline and at 4, 8, and 12 months. Notably, most participants showed significant improvement after eight months, which aligns with prior evidence suggesting that structural and functional brain changes may require sustained therapeutic engagement [70]. These findings support the hypothesis that psychotherapy influences not only symptom profiles but also core neurocognitive systems implicated in BPD, such as error monitoring, social evaluation, and emotional conflict resolution.

These longitudinal effects imply that psychodynamic therapy could be an effective, non-pharmacological approach to neuroplastic adaptation in emotional regulation systems, complementing the mechanistic insights derived from pharmacological and neuromodulatory studies.

### 9.3. Preventive Interventions

Intervention programs targeting high-risk populations, particularly those with a history of early trauma or displaying early behavioral indicators of emotional dysregulation, are crucial in reducing both the prevalence and severity of BPD.

- Parental guidance programs: These interventions facilitate the development of secure attachment relationships and diminish the adverse effects of early childhood trauma on brain structure in the long term [71,72].
- Adolescent interventions: The MOBY (Making Our BPD Youth) trial is a landmark randomized controlled trial evaluating early intervention strategies for adolescents and young adults presenting with borderline personality features. Conducted in Australia, the trial involved 139 participants aged 15–25, comparing 3 conditions: integrated cognitive analytic therapy (ICAT), specialized early intervention using adaptive DBT principles, and a control group receiving general clinical management [53].

The results showed that both active treatments led to significant reductions in symptom severity, self-harm, and functional impairment over 18 months. The ICAT-informed approach produced slightly greater improvements in interpersonal functioning. Importantly, the trial emphasized the feasibility and acceptability of early intervention for young people at risk of BPD, advocating for service models that address identity formation, emotional regulation, and relational capacities beyond merely suppressing symptoms. These findings support the integration of structured psychotherapeutic models into public mental health systems for young people with emerging personality pathology.

### 9.4. Psychoeducation and Empathy Development

Neurobiological studies can help with psychoeducation by informing patients and their families about the biological foundations of BPD, thereby reducing stigma and fostering empathy. Knowing that emotional dysregulation is caused by neural disruption rather than character defects encourages a more compassionate treatment attitude [31,60].

To achieve the best clinical results, future studies should prioritize the following:

- The development of reliable biomarkers to improve early diagnosis and predict treatment outcomes.
- Refining neuromodulation techniques to target particular brain networks implicated in BPD.
- Longitudinal research to determine the association between neurobiological changes and symptom relapse and remission.
- Treatment efficacy: combining biology and psychology for individualized treatment.

## 10. Limitations and Challenges

Despite considerable advances in the neurobiological and psychological features of BPD, there are several common problems and shortcomings in research, practice, and implementation.

### 10.1. Methodological Limitations in Neuroimaging Research

- Small sample sizes: Most neuroimaging research on BPD features small sample sizes, which compromises both the statistical power and external validity of the results. These small samples do not fully capture the heterogeneity of the disorder completely and could lead to inflated effect sizes or the identification of false positives [28].
- Imaging protocol variation: Inconsistencies in neuroimaging protocols, including differences in fMRI conditions and preprocessing procedures, hinder the comparison of results across different studies. Without standardized protocols, it is impossible to integrate findings into unifying models that describe the neurobiological underpinnings of BPD [73].
- Limitations of longitudinal data: Many studies rely heavily on cross-sectional methodology, which restricts our knowledge of the progression of neural alterations in BPD over time. Longitudinal studies are necessary to adequately explain the timing of neurobiological alterations as well as in relation to treatments [74].

### 10.2. Heterogeneity and Co-Morbidities

- Symptom heterogeneity: BPD is highly heterogeneous, with significant variability in symptom manifestation among subjects. This variability poses challenges for identifying specific neural correlates and may require subgroup analyses to better refine neurobiological models [75].
- Comorbid psychiatric disorders: The high prevalence of comorbid disorders, such as depression, PTSD, anxiety, and substance use disorders, makes it difficult to determine BPD-specific neural abnormalities [76]. Neurobiological findings could represent transdiagnostic effects rather than BPD-specific effects.

### 10.3. Ethical Considerations in Research

- Participant vulnerability: Given the high level of trauma and emotional instability characteristic of BPD samples, ethical issues can take on an even greater importance. Researchers must be sensitive to avoid exacerbating psychological distress or retraumatizing participants [77].
- Risk of stigmatization: Research on the neurobiological basis of BPD has the potential to contribute to stigmatization. The findings can be misinterpreted as indicating inherent deficits, thereby reinforcing negative stereotypes about individuals with BPD. Therefore, care should be taken when reporting research findings to enhance understanding and reduce stigma [9].

### 10.4. Challenges in Genetic and Epigenetic Research

- Lack of specificity: Despite research into genetic and epigenetic processes identifying some potential markers, no single marker has been regularly shown to be associated with borderline personality disorder [48]. The interaction between genetic risk factors and other psychiatric disorders makes it difficult to identify clear-cut biomarkers.
- Underpowered and small studies: Most genetic and epigenetic studies are underpowered and small since they include low sample sizes in order to detect subtle yet significant associations [9]. Large well-powered studies are the only ones that can generate proof of validation for promising epigenetic and genetic markers.

### 10.5. Technological and Analytical Limitations

- Complexity of neural networks: The intricacy of brain connectivity, along with how it is modulated by environmental influences, poses significant challenges to current neuroimaging techniques. Advanced methodologies, such as network analysis and machine learning, are required to elucidate the complex neural interactions observed in BPD [78].
- Limited practical application: Translating neurobiological findings into clinical practice remains difficult. Although neuroimaging and genetic findings offer the prospect of personalized medicine, their everyday use is also restricted by costs, access, and the need for specialized expertise [66].
- These challenges can be addressed using the following approaches:
- Large multi-site studies with standardized procedures can enhance the reliability and relevance of neurobiological findings.
- Longitudinal prospective studies to track neural and psychological changes over time, and their relationship to clinical recovery.
- Ethical standards to maintain participant well-being and reduce stigma.
- Cutting-edge analytical methods for investigating complex brain–behavior relationships in BPD.

### 10.6. Addressing Research Challenges

To address these limitations, future research should prioritize large, multicentre studies using standardized imaging and diagnostic protocols to improve replicability and generalizability [73]. Longitudinal and prospective study designs are essential for mapping the trajectory of neurobiological changes in BPD and identifying sensitive windows for intervention [11]. Furthermore, integrating machine learning and advanced network analysis methods could offer more nuanced insights into brain–behavior relationships and assist in stratifying BPD phenotypes based on neurobiological profiles [79]. Finally, ethical research frameworks must be strengthened to ensure participant safety and reduce stigma, particularly when working with trauma-exposed and emotionally vulnerable populations [9,77].

## 11. Discussion

This systematic narrative review summarizes the latest neurobiological research on BPD, providing an integrative framework that links structural, functional, neurochemical, and genetic changes to the disorder’s main clinical symptoms. Our analysis emphasizes the multifactorial nature of BPD, highlighting how neurodevelopmental vulnerabilities and trauma-related epigenetic changes interact with brain-based dysfunctions to influence symptom presentation.

### 11.1. Comparison with Prior Reviews

Our findings are consistent with the previous literature identifying amygdala hyperreactivity and prefrontal hypoactivation as central to BPD pathophysiology [18,28]. However, our work goes further by incorporating recent studies on network-level dysfunctions, such as disrupted default mode network (DMN) activity, mentalising networks, and the mirror neuron system [11,80]. These insights offer a neurobiological explanations for identity diffusion, interpersonal hypersensitivity, and dissociative states, which have traditionally been conceptualised in psychodynamic terms [61].

Unlike earlier meta-analyses [19,65], which emphasised isolated regional changes, our approach highlights connectivity impairments—notably between the prefrontal cortex (PFC) and limbic structures—as a more dynamic and explanatory model for understanding affective instability and behavioral dyscontrol.

### 11.2. Functional and Clinical Interpretation

We interpret the observed brain alterations as developmentally sensitive markers shaped by adverse life experiences, rather than static deficits. For example, hippocampal atrophy is not only linked to trauma exposure, but also to impaired autobiographical memory consolidation and contextual emotion processing [81]. Similarly, weakened PFC–amygdala coupling is interpreted as the neural basis for poor emotional regulation and the persistence of hyperaroused states in emotionally charged situations [82].

The review also highlights neurochemical contributions, including serotonergic dysregulation (linked to impulsivity), dopaminergic imbalance (linked to affective volatility), oxytocin deficits (linked to attachment disturbances), and glutamatergic anomalies (linked to cognitive fragmentation). Each of these offers a specific therapeutic entry point [31,37,83].

### 11.3. Points of Divergence and Methodological Gaps

Discrepancies remain in the literature, particularly regarding dopamine function and genetic susceptibility markers. These discrepancies may reflect heterogeneity in BPD presentation or sampling biases [42]. Furthermore, the field is plagued by methodological limitations, including small sample sizes, cross-sectional designs, and an inability to replicate findings. Moving forward, longitudinal and multimodal neuroimaging studies, especially those integrating trauma histories, psychometric traits, and treatment responses, are urgently needed [84].

### 11.4. Integrative Advances: From Network Disruption to Predictive Biomarkers

Recent studies have advanced our understanding of BPD by integrating functional neuroimaging, machine learning, and longitudinal therapeutic outcomes.

#### 11.4.1. Functional Connectivity and Network Efficiency

Resting-state fMRI data increasingly highlight disrupted connectivity between emotion regulation and self-referential networks in BPD. Leichsenring et al. (2024) [9] summarized the finding of increased amygdala–default mode network (DMN) coupling, reduced integration within fronto–limbic circuits, and altered global efficiency—particularly in such regions as the middle temporal gyrus. These patterns are also linked to personality dimensions (e.g., separation insecurity and depressivity) and may serve as network-level endophenotypes [9].

#### 11.4.2. Machine Learning and Diagnostic Precision

Montgomery (2025) introduced a high-accuracy EEG-based machine learning classifier for BPD diagnosis [17]. This achieved 88% overall accuracy using a multilayer perceptron model that was trained on frontal and temporal spectral features. The most discriminative signals emerged from theta and beta band asymmetries, particularly over the dorsolateral prefrontal cortex. This model suggests a scalable, cost-effective approach to identifying biomarkers that could be used in forensic and clinical psychiatry.

#### 11.4.3. Predictive Modeling of Treatment Response:

In a longitudinal functional magnetic resonance imaging (fMRI) study, researchers used machine learning to predict clinical improvement following dialectical behavior therapy (DBT). Pre-treatment neural activation in the anterior cingulate cortex (ACC), alongside subcortical limbic responses, predicted symptomatic change with up to 72% accuracy [85]. Notably, improvements in reflective functioning and alexithymia were associated with the normalization of dorsal ACC activity at the 12-month follow-up point, offering a neurobiological basis for psychodynamic progress.

## 12. Critical Synthesis and Future Directions

### 12.1. Transdiagnostic Comparisons

Recent neuroimaging research has emphasized the importance of comparing BPD with other clinical populations that also exhibit default mode network (DMN) hyperconnectivity. Both post-traumatic stress disorder (PTSD) and cocaine use disorder (CUD) share patterns of increased functional connectivity within the DMN. However, BPD is characterized by the dominance and stability of hyperconnected precuneus states, which appear to be more independent of other states. This may reflect trait-level dysfunction and suggests potential utility as a differential biomarker. Table 2 summarizes these transdiagnostic overlaps and distinctions, highlighting the shared disruption to salience and emotional regulation networks, as well as disorder-specific patterns in DMN topology. Table 4 provides a comparative summary of DMN hyperconnectivity and related neurofunctional alterations observed in BPD, PTSD, and CUD, highlighting overlapping and distinct patterns across these disorders.

### 12.2. Heterogeneity Within BPD

There is mounting evidence to support the idea that there are neurobiological and behavioral distinctions between internalizing and externalizing subtypes of BPD. Internalizing phenotypes are characterized by heightened emotional instability, social withdrawal, and increased activity in the DMN, whereas externalizing types often exhibit impulsivity, aggression, and hyperactivation in the salience network. These phenotypic profiles influence both neural connectivity and treatment response. We propose that future neuroimaging and therapeutic trials adopt stratified designs that explicitly incorporate this heterogeneity, thereby reducing noise and improving replicability.

### 12.3. Clinical Implications of Classifier Accuracy

A supervised machine learning approach was implemented to classify individuals with BPD based on EEG-derived neurofunctional features. The analysis used data from n = 112 participants and employed a 10-fold cross-validation scheme to optimize generalizability and prevent overfitting. The resulting model achieved an accuracy of 82%, with precision of 0.79 and recall of 0.84, indicating strong performance in identifying neural patterns specific to BPD. These findings highlight the potential of EEG-based machine learning for developing objective, high-accuracy diagnostic tools in forensic and clinical psychiatry. Table 3 illustrates how core neurobiological findings in BPD—spanning structural, functional, and neurochemical domains—can inform clinical decision making in terms of diagnosis, intervention planning, and prevention strategies.

### 12.4. Limitations and Future Directions

Despite substantial progress in understanding the neurobiology of BPD, there are still several methodological, structural, and conceptual limitations that restrict the interpretation and generalizability of current findings.

Firstly, many of the reviewed studies are retrospective in nature, which limits our ability to infer causal relationships between neurobiological alterations and BPD symptoms. Additionally, the modest sample sizes in neuroimaging and genetic studies reduce statistical power and increase the risk of type II errors. A further limitation is the lack of multicenter cohorts with adequate power (N > 100), which remains a major barrier to robust and reproducible conclusions.

A second limitation relates to the heterogeneity of diagnostic criteria, assessment tools, and imaging methodologies. Scanner-to-scanner variability, inconsistent acquisition parameters, and the absence of standardized imaging protocols (e.g., TR = 800 ms and isotropic resolution of less than 2 mm) undermine reproducibility and impede large-scale meta-analyses. Standardizing imaging parameters across sites is essential for data pooling and cross-study comparisons.

Furthermore, few studies have employed effective connectivity approaches, such as dynamic causal modeling (DCM) or Granger causality, which could clarify the directionality of the neural interactions underlying core BPD symptoms. Similarly, the field lacks longitudinal, biomarker-driven studies that could help to track disease progression and treatment response over time.

BPD itself presents substantial clinical heterogeneity, with potential subtypes and phenotypic variants that are rarely considered in empirical research. This further complicates efforts to delineate specific neural correlates and may mask subtype-specific biomarkers. Additionally, psychiatric comorbidities (e.g., PTSD, depression, and ADHD) are often not adequately controlled for, which limits inferences about disorder-specific neural alterations.

Finally, several potential biases must be acknowledged. Reliance on peer-reviewed, English-language publications introduces language and selection bias, potentially excluding valuable non-English or unpublished findings. Publication bias may also skew the literature towards studies that report positive or statistically significant outcomes. The lack of formal risk-of-bias assessments, along with the retrospective design and methodological variability of many of the included studies, means that the results should be interpreted with caution and highlights the need for replication using standardized, prospective designs.

In summary, to advance the field, future research should prioritize the following:

- Large, multicenter studies with harmonized protocols;
- Longitudinal and prospective study designs;
- Stratification by clinical subtypes and comorbidities;
- Greater use of effective and dynamic connectivity analyses;
- Adoption of risk-of-bias assessment frameworks in systematic reviews.

## 13. Conclusions

This review synthesized the available evidence on the neural, neurochemical, and psychosocial correlates of BPD, highlighting convergent disruptions in prefrontal–amygdala regulation, default mode network (DMN) dynamics, and social cognition circuits. The findings suggest that certain markers, such as precuneus hyperconnectivity and dorsal anterior cingulate cortex (dACC) hypoactivity, remain robust across studies and may have transdiagnostic specificity. However, the reliability of these biomarkers is limited by small sample sizes, scanner variability, and lack of replication across cohorts. Future work should prioritize imaging modalities with test–retest reliability >0.6 (e.g., rs-fMRI graph metrics).

Looking forward, we propose the following concrete agenda for the next 2–5 years:

(1) The construction of multimodal biomarker panels integrating functional connectivity profiles with epigenetic markers (e.g., FKBP5 methylation).
(2) Randomized neuromodulation trials (e.g., TMS or tDCS) stratified by network phenotype (e.g., DMN vs. salience-dominant dysconnectivity).
(3) Implementation studies testing the clinical utility of predictive models with ≥70% accuracy for monitoring early treatment response in weeks 6–8.

Addressing these challenges will require harmonized protocols (TR = 800 ms and resolution of <2 mm), cross-site data sharing, and attention to internalizing/externalizing heterogeneity. Only through such integrative efforts can we progress towards precision psychiatry for BPD, based not only on neuroscience but also on scalable, person-centered interventions.

## Figures and Tables

**Table 1:** Epidemiological prevalence of borderline personality disorder (BPD) across different population samples. Clinical samples show significantly higher prevalence rates compared to the general population.

Population Type	Prevalence (%)	Reference
General population (lifetime)	1.6	[3]
General population (12-month)	0.7	[4]
Primary care patients	6.4	[5]
Outpatient psychiatric samples	10.0	[6]
Inpatient psychiatric samples	20.0	[7]

**Table 2:** This table provides a synthesized overview of major neurobiological findings in BPD across structural, functional, chemical, genetic, and cognitive domains, highlighting their clinical relevance and the corresponding literature.

Domain	Key Brain Regions/Systems	Associated Symptoms in BPD	Representative Studies
Structural	PFC, amygdala, hippocampus, parietal cortex	Emotional instability, impulsivity, memory impairments	[18,28]
Functional connectivity	PFC–amygdala, DMN, mentalizing networks	Poor emotion regulation, self-disturbance, social cognition deficits	[11,29]
Neurochemistry	Serotonin, dopamine, glutamate, oxytocin	Impulsivity, dysphoria, interpersonal sensitivity	[30,31]
Genetics/epigenetics	5-HTTLPR, DRD4, NR3C1	Increased vulnerability to trauma, emotional reactivity	[15,32]
Cognitive	Executive function, memory, attention	Planning deficits, dissociation, hypersensitivity	[22,25]

**Table 3:** Translating neurobiological findings into clinical practice. This table outlines how key neural alterations in BPD inform diagnosis, treatment, and prevention strategies.

Neurobiological Feature	Clinical Manifestation	Therapeutic Implication
PFC–amygdala disconnect	Affective lability, reactive aggression	Targeted psychotherapy (DBT), neuromodulation (TMS)
Hippocampal atrophy	Dissociative symptoms, trauma sensitivity	Trauma-informed care, EMDR
Low serotonin/dopamine	Impulsivity, suicidality	SSRIs, pharmacogenetic approaches
Oxytocin dysregulation	Interpersonal dysfunction	Social cognition training, oxytocin-based trials
Epigenetic trauma imprinting	Stress sensitivity	Early intervention, resilience-building programs

**Table 4:** Comparative summary of default mode network (DMN) hyperconnectivity and related neurofunctional features across borderline personality disorder (BPD), post-traumatic stress disorder (PTSD), and cocaine use disorder (CUD).

Condition	DMN Hyperconnectivity	Hyper-Connected Precuneus States	Salience Network Disruption	Emotional Dysregulation	Reversibility with Treatment
Borderline personality disorder (BPD)	Yes	Stable, dominant	Yes	Severe	Partial (e.g., DBT, MBT)
Post-traumatic stress disorder (PTSD)	Yes	Contextual, trauma-linked	Yes	High	Moderate (e.g., trauma therapy)
Cocaine use disorder (CUD)	Yes	Less consistent	Moderate	Moderate	Limited

## Data Availability

No new data were created or analyzed in this study.

## Supplementary Materials

The following supporting information can be downloaded at: https://www.mdpi.com/article/10.3390/biomedicines13071783/s1, Table S1: Quality Assessment Using the JBI Checklist.
