# Supplementary material for: Understanding the Borderline Brain: A Review of Neurobiological Findings in Borderline Personality Disorder (BPD)
Source: Biomedicines. 2025 Jul 21;13(7):1783. doi: 10.3390/biomedicines13071783 (PMC12292566; doi:10.3390/biomedicines13071783)
Supplement: Supplementary file 1 [file biomedicines-13-01783-s001.zip › biomedicines-3691837-supplementary.pdf]

## Supplementary Table S1. Quality Assessment Using the JBI Checklist

| Study ID | 1. Clear inclusion criteria | 2. Setting & subjects described | 3. Valid exposure measurement | 4. Valid condition measurement | 5. Confounders identified | 6. Confounding dealt with | 7. Valid outcome measurement | 8. Appropriate statistics | Total Score (0–8) | Quality Rating |
|---|---|---|---|---|---|---|---|---|---|---|
| Schulze et al., 2019 | 1 | 1 | 1 | 1 | 1 | 1 | 1 | 1 | 8 | High |
| Ruocco et al., 2010 | 1 | 1 | 1 | 1 | 1 | 1 | 0 | 1 | 7 | High |
| Krause-Utz et al., 2014 | 1 | 1 | 1 | 1 | 1 | 1 | 1 | 1 | 8 | High |
| Choe et al., 2018 | 1 | 1 | 1 | 1 | 0 | 0 | 1 | 1 | 6 | Moderate |
| Schmidt et al., 2021 | 1 | 1 | 1 | 1 | 0 | 0 | 1 | 1 | 6 | Moderate |
| Montgomery, 2025 | 1 | 1 | 1 | 1 | 1 | 1 | 1 | 1 | 8 | High |
| Nysæter et al., 2010 | 1 | 1 | 1 | 1 | 1 | 0 | 1 | 1 | 7 | Moderate |

| | | | | | | | | | | |
|---|---|---|---|---|---|---|---|---|---|---|
| Schmahl et al., 2003 | 1 | 1 | 1 | 1 | 1 | 0 | 1 | 1 | 7 | Moderate |
| Michel et al., 2024 | 1 | 1 | 1 | 1 | 1 | 1 | 1 | 1 | 8 | High |
| Perroud et al., 2011 | 1 | 1 | 1 | 1 | 0 | 0 | 1 | 1 | 6 | Moderate |
| Drews et al., 2019 | 1 | 1 | 1 | 1 | 1 | 1 | 1 | 1 | 8 | High |
| Park et al., 2018 | 1 | 1 | 1 | 1 | 1 | 1 | 1 | 1 | 8 | High |
| Zilverstand et al., 2024 | 1 | 1 | 1 | 1 | 1 | 1 | 1 | 1 | 8 | High |
| Visintin et al., 2016 | 1 | 1 | 1 | 1 | 1 | 1 | 1 | 1 | 8 | High |
| Schuermann et al., 2011 | 1 | 1 | 1 | 1 | 0 | 0 | 1 | 1 | 6 | Moderate |
| Salas et al., 2023 | 1 | 1 | 1 | 1 | 0 | 0 | 1 | 1 | 6 | Moderate |
| Lahnakoski et al., 2024 | 1 | 1 | 1 | 1 | 1 | 1 | 1 | 1 | 8 | High |
